# The Influence of 1-Butanol and Trisodium Citrate Ion on Morphology and Chemical Properties of Chitosan-Based Microcapsules during Rigidification by Alkali Treatment

**DOI:** 10.3390/md12125801

**Published:** 2014-12-02

**Authors:** Sudipta Chatterjee, Fabien Salaün, Christine Campagne

**Affiliations:** 1 University of Lille Nord de France, F-59000 Lille, France; E-Mails: sudipto_ch77@yahoo.co.in (S.C.); christine.campagne@ensait.fr (C.C.); 2 ENSAIT/GEMTEX, F-59100 Roubaix, France

**Keywords:** microcapsules, linseed oil, chitosan, 1-butanol, trisodium citrate, oil-in-water emulsion, alkali treatment

## Abstract

Linseed oil which has various biomedical applications was encapsulated by chitosan (Chi)-based microcapsules in the development of a suitable carrier. Oil droplets formed in oil-in-water emulsion using sodium dodecyl sulfate (SDS) as emulsifier was stabilized by Chi, and microcapsules with multilayers were formed by alternate additions of SDS and Chi solutions in an emulsion through electrostatic interaction. No chemical cross-linker was used in the study and the multilayer shell membrane was formed by ionic gelation using Chi and SDS. The rigidification of the shell membrane of microcapsules was achieved by alkali treatment in the presence of a small amount of 1-butanol to reduce aggregation. A trisodium citrate solution was used to stabilize the charge of microcapsules by ionic cross-linking. Effects of butanol during alkali treatment and citrate in post alkali treatment were monitored in terms of morphology and the chemical properties of microcapsules. Various characterization techniques revealed that the aggregation was decreased and surface roughness was increased with layer formation.

## 1. Introduction

Microencapsulation is a useful and promising technology where biologically active agents such as drugs, functional foods, and probiotics are enclosed within a semi-permeable polymer membrane to assist various pharmaceutical and biomedical processes [1,2]. The striking features of microcapsules include sufficient resistance to environmental conditions, permeability, and release properties [3]. Nowadays, microcapsules are successfully being applied in various biomedical and pharmaceutical processes and, in addition to biomedical applications, encapsulated microcapsules have become very effective materials in food, printing, cosmetics, agriculture, and biotechnology applications [4]. Microcapsules are starting to receive attention in textile applications because various active substances, such as phase change materials, fragrances, flame retardant, cooling agent, essential oils, skin moisturizing agents and probiotic organisms [5] may be protected to achieve new smart functionalities of a textile substrate.

Microcapsules’ shell can be made from various polymers, and nanoparticles such as silica and polysiloxane [6]. In particular, natural biodegradable polymers such as chitosan, gelatin, albumin, and alginate are receiving considerable attention due to their biocompatibility and good release properties of the membrane [7,8]. Chitosan (Chi) has been found to be an effective wall material of microcapsules in biomedical fields due to its ability to reside at the target sites within the human body for prolonged periods with controlled release properties [9]. Chi, a natural polycationic compound, is obtained from chitin by alkaline deacetylation, and it shows good solubility in dilute aqueous acid solution because of protonation of the amine groups of glucosamine monomers [10,11]. Chi is also being used as wall materials of microcapsules in food [12], tissue engineering [13], and textile applications [14,15], as it is biodegradable, nontoxic and biocompatible [16]. Chi-based microcapsules obtained by emulsification, precipitation, and spraying methods have single or multilayer membrane structure depending on the microencapsulation method [17,18]. In many scientific literatures, microcapsules are reported to be formed with cationic polyelectrolyte Chi by electrostatic interaction with negatively charged alginate molecules [19], and Chi-alginate microcapsules have been proved to be attractive in multiple fields such as biomedicine, biotechnology, and food [20].

In our earlier study, linseed oil-loaded multilayer microcapsules were developed by oil-in-water emulsification using sodium dodecyl sulfate (SDS) as anionic emulsifier and then, formation of multilayer shell membrane was done through ionic gelation by stepwise addition of the membrane materials Chi and SDS in the emulsion [21]. Linseed oil is obtained from the seed of the flax plant *Linum usitatissimum* and, in comparison with other vegetable oils, linseed oil is distinguished by having the highest content of α-linolenic acid (52% of total fatty acids) which is reported to have a role in decreasing inflammation [22]. Natural polyesters formed from linseed oil have several applications, including biodegradable elastomers and adhesives [23]. The linseed oil-loaded microcapsule formation involved electrostatic interaction between SDS and Chi on the surface of oil droplets stabilized by SDS molecules in the emulsion [24,25]. Biomedical application of SDS is already reported and the vesicles formed with SDS and positively charged azobenzene containing surfactant are used in developing a gene delivery system where photo-triggered release of nucleic acids from the vesicles is achieved [26,27]. Mild alkali treatment of microcapsules was done to solidify the outer shell of microcapsules and charge neutralization of amine groups of Chi on the microcapsules by alkali led to the formation of flocculi in the suspension [21]. However, the post treatment of microencapsules with alkali gave rise to some undesired gel formation by Chi chains and flocculi developed from alkali treatment often produced aggregates in the system, and this is the main drawback for commercial use of this slurry in various applications especially for textiles.

In the present study, linseed oil loaded microcapsule samples obtained from step by step process using Chi and SDS solutions were subject to alkali treatment with a small amount of 1-butanol (butanol) to avoid aggregate formation during alkali treatment. In the study, no chemical cross-linker was used to rigidify the shell membrane and rather ionic gelation between Chi and SDS was applied to develop consecutive shells. Butanol, a primary alcohol with a 4-carbon structure was used in this study to retard gel formation by Chi chains in the system. It was reported in literature that swelling of Chi hydrogel was less in a butanol-water mixture than only in water due to the hydrophobic character of alkyl group (butyl) attached to the hydroxyl group of the alcohol [28]. A small amount of butanol was added in alkali solution to restrict swelling of the outermost shell of microcapsules. In this study, alkali treated microcapsule samples were subject to react with trisodium citrate (citrate) solution for buffering action and ionic cross-linking. Rana* et al.* reported [29] that cationic polyamines like Chi attain ordered microcapsule structure under certain salt solutions, and multivalent counter-ion condensation of Chi in citrate solution leads to rapid formation of ordered microcapsules structure from ionically cross-linked polymer aggregates. Chi ionically cross-linked with citrate shows pH dependent swelling in gastro-intestinal environment [30,31]. The zeta potential and size distribution of alkali-treated samples were investigated to understand the effect of butanol stabilization and citrate treatment on the microcapsules. The changes in morphology and properties of alkali treated microcapsule samples were monitored using FTIR, optical microscopy, scanning electron microscopy (SEM), atomic force microscopy (AFM) combined with surface roughness, and wetting measurements.

## 2. Results and Discussion

### 2.1. Step by Step Microencapsulation Process and Alkali Treatment of Microcapsules

The microcapsules were formed by deposition of Chi on the surface of oil droplets in an oil-in-water emulsion using SDS as anionic emulsifier. Linseed oil was encapsulated by Chi based microcapsules to develop carrier system for this flaxseed oil which has various pharmacological applications [32]. The interaction of positively charged Chi molecules with anionic SDS molecules on the surface of oil droplets led to the microcapsules’ shell formation in the emulsion system [21].

The multilayer membrane formation on the microcapsules involved alternate step-by-step addition of SDS and Chi solutions. The rearrangement of the charge groups of wall materials (Chi and SDS) on the surface took place in order to add layers by electrostatic interaction during a step-by-step addition process. However, the electrostatic interaction between SDS and Chi on the surface of microcapsules was partially hindered by the large hydrophobic part of wall materials and this resulted in lowering of interaction between oppositely charged groups of wall materials on the surface of microcapsules [33]. Moreover, unwanted complex formation between Chi and SDS molecules was increased during multilayer microcapsule formation. In our earlier publications, we reported strategies to reduce aggregate formation and enhance interaction of wall materials during formation of microcapsules with a multilayer shell structure [15,33]. The formation of microcapsules by step by step addition of Chi and SDS solutions gave rise to multilayer membrane structure, and positive zeta potential values of microcapsules indicated that microcapsules were formed with a high residual positive charge on their surface [21]. Additionally, positive zeta potential values of microcapsules were found to be increased with layer numbers (≥+30 mV) as microcapsules were becoming overcharged due to the positive charge of Chi molecules. The overcharging of microcapsules’ surfaces with positive charge of Chi molecules impeded successive layer formation as SDS addition during multilayer formation could not switch the zeta potential value of microcapsules from positive to negative [21]. So, the calculated amount of SDS and Chi solutions was added during multilayer shell formation in order to avoid overcharging by Chi moieties and maximize the interaction between oppositely charged wall materials [15,33].

The mild alkali treatment of microcapsules was done in order to rigidify the outermost shell of microcapsules. The addition of mild NaOH solution (0.02 N) to microcapsules’ suspension led to the development of flocculi by charge neutralization of amine groups of Chi in the microcapsules, and flocculation indicated the phase separation of linseed oil-loaded microcapsules after alkali treatment in the suspension. Nevertheless, alkali treatment of microcapsules was always accompanied by gel formation of Chi molecules and aggregation of microcapsules as charge neutralization took place at protonated amine groups of Chi on the surface of microcapsules by alkali solution. The addition of a small amount of butanol in alkali solution during alkali treatment of microcapsules reduced unwanted gel formation by Chi molecules and impeded aggregate formation by microcapsules in microcapsule suspension.

### 2.2. Influence of Butanol Stabilization on Alkali Treatment of Microcapsules

The property and morphology studies were carried out for a one-layer microcapsule sample to investigate first shell membrane formation over oil droplets in an oil-in-water emulsion, four layers to understand multilayer formation, and 10 layers to study the final product. As shown in Table 1, the mean diameter of alkali treated microcapsules with butanol stabilization varied according to the number of layers of microcapsules. The mean diameter values of alkali-treated microcapsule samples without butanol stabilization were reported earlier, and the mean diameters ± SD of one layer, four layers and 10 layers were 3.6 μm ± 3.4, 5.1 μm ± 5.1, and 5.4 μm ± 4.5, respectively [21]. Table 1 clearly indicates that mean diameter of microcapsules was significantly reduced after butanol treatment up to four layers. The mean diameters of alkali-treated microcapsules after butanol treatment were also measured for six layers (37.5 μm ± 17.0) and eight layers (54.1 μm ± 20.5). The earlier reported mean diameters values of six layers (6.0 μm ± 5.6) and eight layers (6.5 μm ± 4.9) without butanol treatment indicated that mean diameter values of alkali treated microcapsules (four, six and 10 layers) after butanol treatment were higher than samples without butanol treatment. Butanol showed a stabilizing effect during alkali treatment to reduce the aggregation of the microcapsules by controlling their swelling in mild aqueous alkali solution up to four layers [28]. The increased diameter of the sample after four layers might be explained by enhanced swelling of microcapsules in the alkali solution as close packing of the multilayer was somehow relaxed to increase its mean diameter.

The alkali-treated one-layer microcapsules showed negative zeta potential of −12.3 mV, whereas alkali-treated microcapsule samples with higher layer numbers showed positive zeta potential values with butanol treatment (Table 1). The zeta potential values were found to be very similar to the earlier reported values for alkali-treated samples before butanol stabilization [21]. The zeta potential values of alkali-treated microcapsules without butanol treatment were reported to be −20.4, +3.6, and +28.5 mV for one layer, four layers and 10 layers, respectively [21]. The zeta potential results indicated that added alkali amount to the microcapsules suspension was not enough for complete charge neutralization of microcapsules after one layer.

**Table 1:** Mean diameter, zeta potential, and pH values of alkali treated microcapsule samples after butanol stabilization and trisodium citrate treatment.

Microcapsule Samples	Alkali Treated Samples ^a^			Alkali Treated Samples ^b^		
	Mean Diameter (μm)	Zeta Potential (mV)	pH	Mean Diameter (μm)	Zeta Potential (mV)	pH
1 layer	2.3 ± 0.8	−12.3	5.4	3.3 ± 1.1	−1.8	7.0
4 layers	4.5 ± 2.5	+7.7	5.4	5.4 ± 4.2	−1.7	7.0
10 layers	61.5 ± 5.2	+19.5	5.4	69.7 ± 18.8	−2.3	7.0

^a^ Butanol stabilization; ^b^ Butanol stabilization & trisodium citrate treatment ± Standard deviation (SD).

**Figure 1:**
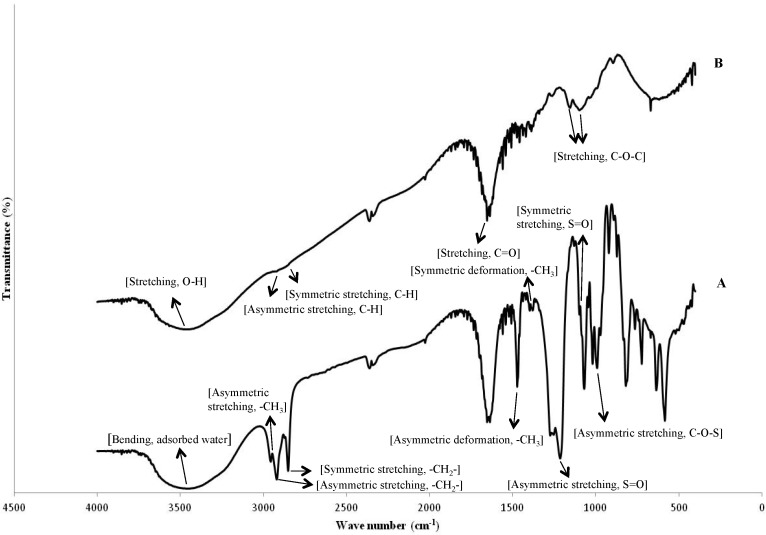
FTIR spectra of (**A**) sodium dodecyl sulfate (SDS); (**B**) low molecular weight Chi.

Microcapsules were formed from an oil-in-water emulsion using Chi and SDS as shell materials. FTIR spectra of Chi (Sigma-Aldrich, Saint-Quentin Fallavier, France) and SDS were given in Figure 1. SDS powder (Figure 1A) showed peak near 3450 cm^−1^ representing the bending vibration of adsorbed molecular water. A peak at 2950 cm^−1^ indicated the asymmetric stretching vibration of -CH_3_, and peaks assigned to asymmetric and symmetric stretching of -CH_2_- were found at 2920 and 2850 cm^−1^, respectively. The asymmetric and symmetric deformation vibrations of -CH_3_ were found at 1470 and 1380 cm^−1^, respectively. The peaks for asymmetric and symmetric stretching of the S=O of SDS were found at 1220 and 1108 cm^−1^, respectively. The peak at 995 cm^−1^ was assigned to the asymmetrical stretching vibration of C–O–S. FTIR spectra of low molecular weight Chi powder showed its characteristic peaks: O-H and N-H stretching of Chi powder near 3470 cm^−1^; asymmetric and symmetric stretching of C-H of Chi at 2928 and 2856 cm^−1^, respectively; C-O stretching of amide I at 1652 cm^−1^, and C-O-C stretching of glycosidic linkage at 1146 and 1096 cm^−1^.

**Figure 2:**
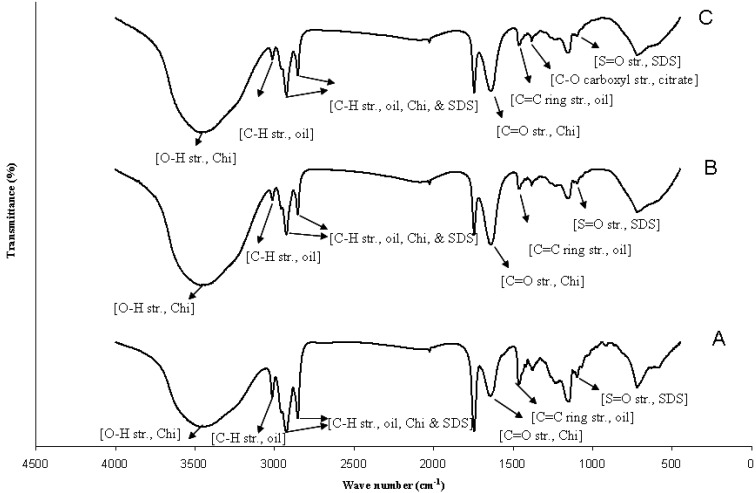
FTIR spectra of (**A**) alkali treated microcapsule sample; (**B**) alkali treated microcapsule sample with butanol stabilization; **C**: alkali treated microcapsules sample with butanol stabilization and citrate treatment.

Linseed oil-loaded alkali-treated microcapsule samples after butanol stabilization showed characteristic FTIR peaks of the components (Figure 2), 3476 cm^−1^ representing O-H and N-H stretching of Chi; 2926 and 2857 cm^−1^ representing C-H stretching (asymmetric and symmetric, respectively) of CH_2_ for linseed oil, Chi, and SDS; and the shoulder on 2926 cm^−1^ at 2956 cm^−1^ represented asymmetric C-H stretching of CH_3_. The peak at 3014 cm^−1^ represented C-H stretching of aliphatic -CH=CH- which was related to un-conjugated cis-double bonds of linseed oil [34]. The peak at 1648 cm^−1^ was representing C=O stretching of amide I of Chi. The other peaks were at 1556 cm^−1^ (N-H bending of amide II of Chi), 1464 cm^−1^ (C=C ring stretching of linseed oil), and 1106 cm^−1^ (S=O stretching of SDS). FTIR spectra of alkali-treated samples before butanol stabilization were listed in Figure 2 and the spectral characteristics of microcapsules were not noticeably changed after butanol addition during alkali treatment as butanol only acts as a stabilizer in the system.

**Figure 3:**
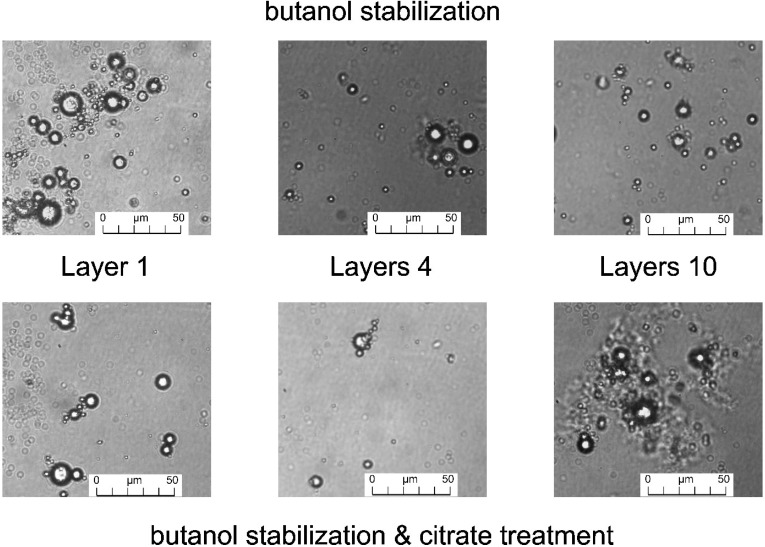
Optical microscope images at 40× of alkali treated microcapsule samples with butanol stabilization: (**top**) and alkali treated microcapsules samples with butanol stabilization and citrate treatment (**bottom**).

The optical microscopy of alkali-treated microcapsules after butanol treatment (Figure 3) at 40× magnification indicated that microcapsules were well dispersed in the suspension, and sample with 10 layers (Figure 3) showed highly dispersed microcapsules in the micrographs. The optical micrographs exhibited that the unwanted gel formation and aggregation of microcapsules were highly decreased by butanol addition during alkali treatment. Thereby, butanol as a stabilizer during alkali treatment of microcapsules inhibited aggregate formation and acted as dispersant to disperse microcapsules in the system.

**Figure 4:**
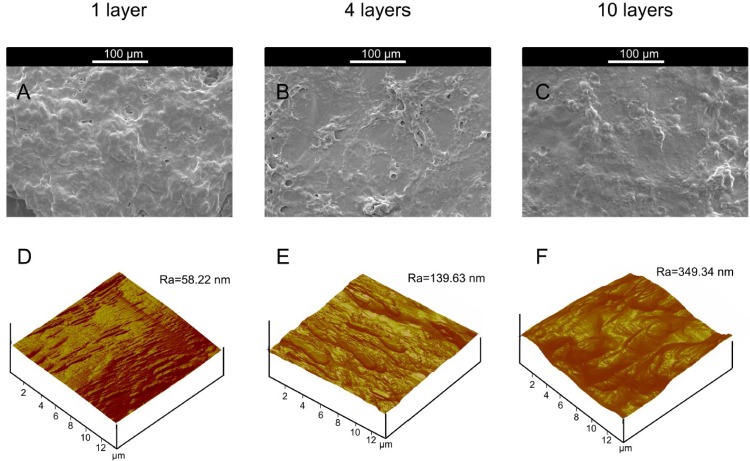
SEM images at 300× of alkali treated microcapsule samples with butanol stabilization: (**A**) one layer; (**B**) four layers; (**C**) 10 layers; AFM images of alkali-treated microcapsule samples with butanol stabilization: (**D**) one layer; (**E**) four layers; (**F**) 10 layers.

The formation of film or functional coating from microcapsule samples over drying on solid support occurred in two consecutive steps: at first, coalescence of the particles occurred followed by a drying step in which the water was released to rigidify or dehydrate the polymer film. It should be taken into account that the shell of microcapsules was not rigidified using any chemical cross-linker and the microcapsule suspension did not produce solid powder after drying on substrate at room temperature. Therefore, the influence of the alkali treatment on the microcapsules could be detected from the surface modification, characterized in this study by roughness, and surface energy of functional coating. The SEM image of functional coating formed from the butanol stabilized one-layer sample (Figure 4A) at 300× magnification showed the presence of microcapsules among a considerable amount of free polymer (Chi). The SEM images of four layers (Figure 4B) and 10 layers (Figure 4C) at 300× indicated the presence of very few microcapsules in a significant amount of polymers, and aggregates were found in their respective functional coatings. Thereby, post treatment of microcapsules caused significant changes on the surface of functional coating, and aggregate formation after post treatment was higher for the microcapsules with a higher number of layers.

As seen in Table 2, the γ*^p^* value of functional coating formed from butanoyl-stabilized alkali-treated microcapsules was increased with an increase in layer numbers, and the γ*^p^* value of four layer-(52.2 mN·m^−1^) samples was much higher than that of one layer (21.0 mN·m^−1^) suggesting that polar functional groups of Chi were more exposed on the surface of four layers than that of one layer. Furthermore, a lower γ*^d^* value of four layers (15.0 mN·m^−1^) than that of one layer (26.6 mN·m^−1^) was also obtained. These surface energy values suggested that arrangement of polar groups on the surface of microcapsules took place. The lower γ*^p^* values of functional coatings for 10 layers (33.9 mN·m^−1^) than that of four layers (52.2 mN·m^−1^), and also, higher γ*^d^* values of 10 layers (32.2 mN·m^−1^) than that of four layers (15.0 mN·m^−1^) emphasized that polar groups of Chi were found less on the surface of functional coating with higher layer numbers. During consecutive layer formation on the shell membrane, the hydrophobic non polar groups of SDS and Chi were found more on the surface than that of polar groups of shell materials and that resulted in higher γ*^d^* values of 10 layers than that of four layers, and lower γ*^p^* values of 10 layers than that of four layers as well. In general, the masking of polar amine groups of Chi was found to be lower in the microcapsule sample with found layers and that gave rise to a maximum polarity value for the four layer sample. So, surface energy values of these microcapsule samples indicated that molecular arrangement on the surface of microcapsules took place due to post treatment of microcapsules by alkali with butanol.

As found in Table 2, average surface roughness of functional coating increased with increasing layer numbers of microcapsules, and surface roughness of functional coating was determined from their respective AFM images (Figure 4D–F). The functional coating (one layer) obtained from post treated microcapsules with butanol showed average surface roughness of 58.22 nm (Figure 4D) and this value was increased to 349.34 nm after increasing layer numbers of microcapsules to 10 layers (Figure 4F). Thereby, post treatment with butanol significantly affected the surface roughness of functional coating, and higher surface roughness resulted from uneven distribution of wall materials on the surface during post treatment.

**Table 2:** Contact angle (θ) and surface energy (γ) of alkali treated microcapsule samples with butanol stabilization by the Owens and Wendt method, and mean surface roughness of samples in solid state by AFM analysis.

Alkali Treated Microcapsules Samples (Butanol Stabilization)	θ_water_^a^ (°)	θ_diiodomethane_^a^ (°)	γ*^p^* (mN/m)	γ*^d^* (mN/m)	γ (mN/m)	Ra *^b^* (nm)
1 layers	55.8	46.6	21.0	26.6	47, 6	58.22
4 layers	23.6	60.5	52.2	15.0	67,2	139.63
10 layers	29.4	25.5	33.9	32.2	66,1	349.34

^a^ Average of contact angle of 5 liquid drops; ^b^ Average of surface roughness (*R*) obtained at 10 different locations of AFM image.

### 2.3. Influence of Citrate Treatment on Butanol Stabilized Alkali-Treated Microcapsules

Table 1 showed that alkali treated microcapsules after citrate treatment exhibited similar zeta potential values for all the samples namely, one, four, and 10 layers. The negatively charged citrate ions acted as a conjugated base of weak acid (citric acid) in the suspension and caused same extent of charge neutralization for all alkali-treated microcapsule samples. Thereby, buffering action of citrate was exhibited in the system and that created similar negative zeta potential values for all the alkali-treated microcapsule samples. Moreover, Chi chains were ionically cross-linked by citrate ions that led to rapid formation of ordered microcapsule structure in citrate salt solutions by multivalent counter-ion condensation [29]. The mean diameter results of citrate-treated microcapsules indicated that mean diameter was increased with the number of layers of microcapsules.

Microcapsules after butanol stabilization and citrate treatment showed characteristic FTIR peaks which were very similar to those found for the alkali-treated microcapsule samples before citrate treatment as mentioned above (Figure 2). The peak at 1386 cm^−1^ represented C-O symmetric vibration of carboxyl groups of citrate [35].

The optical microscope images of butanol stabilized alkali treated microcapsules (Figure 3) at 40× magnification showed that microcapsules were well dispersed in the suspension after citrate treatment and the images were very similar to the images found for alkali-treated microcapsules with butanol stabilization (Figure 3). Therefore, no significant change was imposed on the structure of microcapsules by citrate treatment of alkali-treated microcapsules stabilized with butanol. However, some aggregates of microcapsules were found in the suspension of 10 layers (Figure 3), due to charge neutralization and the cross-linking effect of citrate ions.

**Figure 5:**
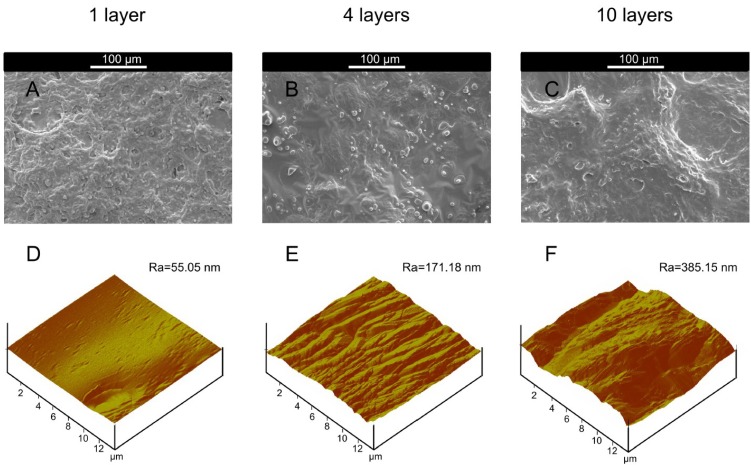
SEM images at 300× of alkali treated microcapsule samples with butanol stabilization and citrate treatment: (**A**) one layer; (**B**) four layers; (**C**) 10 layers; AFM images of alkali treated microcapsule samples with butanol stabilization and citrate treatment: (**D**) one layer; (**E**) four layers; (**F**) 10 layers.

SEM images of alkali-treated microcapsule samples after butanol stabilization and citrate treatment (Figure 5A–C) showed that one layer sample at 300× (Figure 5A) had the presence of few microcapsules among a substantial amount of free polymer. SEM study was done for functional coating which was developed from microcapsule suspension after drying the substrate at room temperature, and the functional coating was formed from free Chi chains due to water evaporation. SEM micrographs showed that some microcapsules were embedded in the functional coating formed by free Chi chains. SEM analysis of functional coating of four layers (Figure 5B) and 10 layers (Figure 5C) after citrate treatment showed the presence of few microcapsules among a significant amount of polymer. Thereby, the morphology obtained from SEM analysis clearly indicated that the citrate treatment of the samples could not impart any significant effect on the morphology of the materials as the samples were more or less the same.

Table 3 showed that γ*^p^* value of four layers (36.4 mN·m^−1^) after citrate treatment of butanol stabilized alkali treated microcapsules was higher than that of one layer (20.3 mN·m^−1^), and this trend clearly indicated that microcapsules with four layers possessed more polar functional groups of Chi on the surface that of one layer. However, four layer sample before citrate treatment showed a higher γ*^p^* value (52.2 mN·m^−1^) that that of four layer-samples (36.4 mN·m^−1^) after citrate treatment, and this trend clearly indicated that citrate treatment caused some changes in the arrangement of polar groups of Chi on the surface of microcapsules. Furthermore, γ*^p^* values of 10 layers (33.9 and 31.9 mN·m^−1^) before and after citrate treatment, respectively, suggested that citrate treatment caused some changes in the arrangement of polar groups of Chi on the surface of functional coating formed after drying the sample at room temperature and polar groups of Chi were less exposed on the surface of the samples. This was also confirmed by γ*^d^* values of the samples before and after citrate treatment. Therefore, citrate ions played a role in the arrangement of polar groups of Chi on the surface of microcapsules.

Table 3 clearly indicated that average surface roughness of the samples after citrate treatment was increased by increasing layer numbers and these results were similar to those obtained for the samples before citrate treatment (Table 2). The average surface roughness of the microcapsules with one layer obtained from AFM (Figure 5D) was 55.05 nm, while that of 10 layers (Figure 5F) was 385.15 nm, indicating multilayer formation on the microcapsules enhanced surface roughness of sample. The citrate treatment of butanol stabilized alkali treated microcapsule sample did not impart any additional effect on the surface roughness of functional coating as the values were more or less same for the samples before and after citrate treatment, and these results were highly corroborated by other microscopic observations.

**Table 3:** Contact angle (θ) and surface energy (γ) of alkali-treated microcapsule samples with butanol stabilization and trisodium citrate treatment by the Owens and Wendt method, and mean surface roughness of samples in solid state by AFM analysis.

Alkali Treated Microcapsules Samples (Butanol Stabilization + Citrate Treatment)	θ_water _^a^ (°)	θ_diiodomethane _^a^ (°)	γ*^p^* (mN/m)	γ*^d^* (mN/m)	γ (mN/m)	Ra *^b ^*(nm)
1 layers	55.4	43.1	20.3	28.4	48.7	55.05
4 layers	38.9	51.3	36.4	21.3	57.7	171.78
10 layers	37.1	33.3	31.2	30.0	61.2	385.15

^a^ Average of contact angle of 5 liquid drops; ^b^ Average of surface roughness (*R*) obtained at 10 different locations of AFM image.

## 3. Experimental Section

### 3.1. Materials

Chi having low molecular weight (molecular weight = 50,000–190,000 and deacetylation = 75%–85%), SDS, and linseed oil were purchased from Sigma-Aldrich Co. LLC (Saint-Quentin Fallavier, France). The other analytical grade chemical reagents such as acetic acid, hydrochloric acid, sodium hydroxide, diiodomethane were obtained from Sigma-Aldrich Co. LLC. 1-Butanol and trisodium citrate were purchased from Prolabo, France.

### 3.2. Preparation of Microcapsules

The formulation of microcapsules from oppositely charged Chi and SDS as membrane materials involved step-by-step deposition combined with oil-in-water emulsification process using SDS as an anionic emulsifier. It started with making oil-in-water emulsion by homogenizing 20 wt% linseed oil (Ultra-Turrax, T-25 basic, IKA^®^WERE, Staufen, Germany) with 80 wt% of aqueous SDS (10 g·L^−1^) solution for 30 min at 16,000 rpm and 50 °C. The first step of the step-by-step formation of microcapsules with Chi and SDS involved addition of 35 mL of Chi solution (3%, w/v in 2%, v/v acetic acid) to 100 mL of emulsion containing 8 g·L^−1^ of SDS, and the reaction was carried out under homogenization condition at 50 °C and 16,000 rpm for 15 min. The measured pH of the prepared microcapsules suspension with one layer was found to be 4.2.

The step-by-step microencapsulation process involved alternate addition of 40 mL of 10 g·L^−1^ SDS solution and 20 mL 3% (w/v) Chi solution (50 °C and 1500 rpm) at 30 min time interval between the additions, and the process was repeated 10 times to develop microcapsules with ten layers. The pH of microcapsules suspension during step-by-step layer formation was maintained at pH 4.2 using 0.1 N HCl or 0.1 N NaOH solution.

### 3.3. Modification of Alkali Treatment Step of Microcapsule Suspension

The alkali treatment of microcapsules was done by mixing 5 mL of microcapsule suspension with 20 mL of 0.02 N NaOH at 30 °C for 10 min under stirring at 1500 rpm [20]. In the present study, 1 mL of butanol was added to the alkaline solution to stabilize microcapsules by impeding gel formation of the Chi molecules during alkali treatment and reducing aggregation of microcapsules. The alkali treatment of microcapsules was accompanied by a pH change of the system from pH 4.2–5.4. The microcapsule samples namely, one layer, and four and ten layers were selected for alkali treatment. The microcapsule samples obtained after alkali treatment were subject to citrate treatment. The citrate treatment of alkali-treated microcapsules was done by reacting 25 mL 0.1 (M) citrate solution with 25 mL of alkali treated microcapsules suspension under stirring at 1500 rpm and 30 °C for 30 min. The citrate treatment helped to increase the pH value of the microcapsule suspension from 5.4–7.2 for one layer, and from pH 5.4–7.0 for four, and ten layers as shown in Table 1. Before freeze drying of the sample, repeated washing and centrifugation were done to remove unreacted materials (Chi, SDS, alkali, citarte) and butanol.

### 3.4. Characterization

#### 3.4.1. Zeta Potential Measurement

The zeta potential of alkali-treated microcapsules suspensions was measured by Zetasizer 2000, Malvern instruments Ltd., Malvern, UK after diluting the samples with de-ionized water 1000 times.

#### 3.4.2. Size Distribution Analysis by Granulometry

The size distribution analysis (granulometry) of microcapsules suspension was performed using Accusizer Particle Sizing Systems (770 Optical Particle Sizer, and 770A Autodiluter), Santa Barbara, CA, USA after diluting the sample 1000 times in de-ionized water to measure the mean diameter of microcapsules.

#### 3.4.3. Chemical Characterization

FTIR spectroscopy of freeze dried microcapsule samples was done using Nicolet Nexus FTIR spectrometer. The optical microscopy of microcapsule samples was done using Axioskos Zeiss equipped with a camera (IVC 800 12S). For FTIR analysis of each sample, 3 scans were assigned for the wave number of 4000–450 cm^−1^.

#### 3.4.4. Scanning Electron Microscopy (SEM) and Atomic Force Microscopy (AFM)

The scanning electron microscopy (SEM) of samples was done by Leica Cambridge S-360 microscope (Leica Cambridge Instrument, Cambridge, UK) operated at an acceleration voltage of 20 kV. The samples for SEM were prepared by depositing single drop of a pre-agitated suspension onto carbon tape, followed by drying at room temperature for 48 h. The drying of microcapsule samples on the solid support at room temperature produces a functional coating.

The surface roughness of microcapsule samples in solid state and PET fabric samples was determined by atomic force microscopy (AFM) at ambient conditions using light tapping mode (TM-AFM), Nanoscope III digital instrument (version 3.2) equipped with image processing software, version 3.2 (Digital Instrument Inc., Digital Instrument Inc., Tonawanda, NY, USA). The set point frequency of the silicon pyramidal cantilever with 4–6 Hz scan speed was about 272 Hz. The microcapsule samples used for AFM in the dry state were prepared by making a film on small and thin glass substrate. The mean roughness (Ra) of surface is expressed by the equation:

(1) $$Ra=\frac{1}{L_{x}L_{y}}\int_{0}^{L_{x}}\int_{0}^{L_{y}}\left|F(x,y)\right|dxdy$$

where *L_x_* and *L_y_* are the dimensions of surface, and *F*(*x*, *y*) is the roughness curve relative to the center plane. The mean roughness is the average of Ra obtained at 10 different locations.

#### 3.4.5. Wetting Measurement

The surface energy of microcapsule samples was determined from contact angle values of sample with two different probe liquids by the Owens and Wendt method [36]. The contact angles of microcapsule samples in solid state were determined with GBX Digidrop Contact Angle meter (GBX, Bourg de Peage, France) by sessile drop technique. The Owens and Wendt method is based on the following equations:

(2) $$\gamma_{L}(1+\cos\theta )=2\sqrt{\gamma_{S}^{d}\gamma_{L}^{d}}+2\sqrt{\gamma_{S}^{p}\gamma_{L}^{p}}$$

(3) $$\gamma_{S}=\gamma_{S}^{d}+\gamma_{S}^{p}$$

where θ is contact angle, and γ, γ*^p^*, and γ*^d^* are total, polar component and dispersive component of surface energy, respectively. The two test liquids were water (γ = 72.8 mN·m^−1^, γ*^p^* = 51.0 mN·m^−1^, and γ*^d^* = 21.8 mN·m^−1^) and diiodomethane (γ = 50.8 mN·m^−1^, γ*^p^* = 2.3 mN·m^−1^, and γ*^d^* = 48.5 mN·m^−1^). The subscripts L and S denote liquid and solid, respectively. The samples of microcapsules for surface energy analysis were prepared by uniform deposition of solutions containing microcapsules on thin glass substrate, dried at room temperature conditions for 48 h.

## 4. Conclusions

Linseed oil, a leading source of omega-3 fatty acid α-linolenic acid, was encapsulated by Chi-based microcapsules, and the present study was focused on the development of a suitable carrier system for linseed oil which has various biomedical applications. Multilayer microcapsules were formed from an oil in water emulsification by step wise addition of Chi and SDS solution, and alkali treatment of microcapsules was done to rigidify its outermost shell. In this study, no chemical cross-linker was used to rigidify the shell membrane, and multilayer shell membrane was formed through ionic gelation using SDS and Chi. Rigidification of the shell membrane was finally achieved using a mild alkali solution. However, aggregation of microcapsules and gel formation of Chi molecules in alkali solution were the major drawbacks of the process. The present study was focused on the development of microcapsules with less aggregation during alkali treatment. Here, butanol was used as a stabilizer in alkali solution to minimize the problems of microcapsule aggregation and formation of Chi gel in the system. The changes in morphology and the chemical properties of alkali-treated microcapsules after using butanol were investigated by various microscopic techniques like optical microscopy, SEM and AFM, and analytical techniques like zeta potential, size distribution, FTIR, and wetting measurements. After alkali treatment of microcapsules with butanol, the microcapsules were treated with a citrate solution to stabilize the charge of microcapsules. The citrate ion as a conjugated base of the weak acid (citric acid) caused charge neutralization of protonated amine groups of Chi by buffering action, and all of the microcapsules varieties showed similar zeta potential values. Also, multivalent counter-ion condensation of Chi chains in citrate solutions led to the rapid formation of an ordered microcapsule structure. Citrate treatment did not have any additional stabilizing effect on the structure after butanol but caused some changes in the arrangement of polar groups of Chi on the surface of microcapsules as obtained from wetting experiments. The surface roughness was significantly increased with multilayer formation as the wall materials were not arranged on the surface in the regular manner. The main focus of this study was to develop a carrier system for linseed oil which was formed with better morphological and chemical properties than our earlier reported carrier for linseed oil. In future research, loading and release of linseed oil from the Chi-based carrier and its thermal acid under gastro-intestinal conditions, oxidative (peroxide and anisidine value), and colloidal stability will be reported to give more insight to this carrier for linseed oil.
